# The relation between body mass index and musculoskeletal symptoms in the working population

**DOI:** 10.1186/1471-2474-14-238

**Published:** 2013-08-12

**Authors:** Laura Viester, Evert ALM Verhagen, Karen M Oude Hengel, Lando LJ Koppes, Allard J van der Beek, Paulien M Bongers

**Affiliations:** 1 Department of Public and Occupational Health, the EMGO Institute for Health and Care Research, VU University Medical Center, Amsterdam, The Netherlands; 2 Body@Work, Research Center on Physical Activity, Work and Health, TNO-VU/VUmc, Amsterdam, The Netherlands; 3 Netherlands Organisation for Applied Scientific Research TNO, Hoofddorp, The Netherlands

**Keywords:** Musculoskeletal disorders, Overweight/obesity, Physical workload, Worker population

## Abstract

**Background:**

The primary aim of this study was to investigate the association between BMI and musculoskeletal symptoms in interaction with physical workload. In addition, it was aimed to obtain insight into whether overweight and obesity are associated with an increase in occurrence of symptoms and/or decrease in recovery from symptoms.

**Methods:**

Based on a large working population sample (n = 44,793), using the data from The Netherlands Working Conditions Survey (NWCS), logistic regression analyses were carried out to investigate the association between BMI and musculoskeletal symptoms, with adjustment for potential confounders. Longitudinal data from the Netherlands Working Conditions Cohort Study (NWCCS) of 7,909 respondents was used for the second research aim (i.e., to investigate the transition in musculoskeletal symptoms).

**Results:**

For high BMI an increased 12-month prevalence of musculoskeletal symptoms was found (overweight: OR 1.13, 95% CI: 1.08-1.19 and obesity: OR 1.28, 95% CI: 1.19-1.39). The association was modified by physical workload, with a stronger association for employees with low physical workload than for those with high physical workload. Obesity was related to developing musculoskeletal symptoms (OR 1.37, 95% CI: 1.05-1.79) and inversely related to recovery from symptoms (OR 0.76, 95% CI: 0.59-0.97).

**Conclusion:**

BMI was associated with musculoskeletal symptoms, in particular symptoms of the lower extremity. Furthermore, the association differed for employees with high or low physical workload. Compared to employees with normal weight, obese employees had higher risk for developing symptoms as well as less recovery from symptoms. This study supports the role of biomechanical factors for the relationship between BMI and symptoms in the lower extremity.

## Background

Musculoskeletal disorders (MSDs) represent a considerable health problem in the working population, with low back pain (LBP) as one of the most common MSDs [1]. MSDs have a high impact on the individual worker, due to problems such as pain and limitations in daily activities. Moreover, it has consequences at society level, including employers, as MSDs have been identified as the most common cause of absenteeism from work and work disability [2] and generate high impact on healthcare costs and on costs due to productivity loss in particular [3-5]. As MSDs have a high impact for the individual as well as for society, it is important to gain insight in the risk factors of such disorders in order to find opportunities for prevention.

The origin of MSDs is complex and multi-factorial. Amongst various risk factors, such as heavy lifting [6] and high job demands [7-9], it has been suggested that high body mass index (BMI) (overweight and obesity) might be an independent risk factor for MSDs. To date, the relationship between BMI and MSDs has mainly been investigated in studies on LBP [10]. These cross-sectional and cohort studies showed that overweight and obesity were associated with LBP [10]. While this relationship has been suggested, it could also be argued that BMI is associated with MSDs in other body regions. For symptoms of neck/shoulder, upper and lower limbs, evidence was also found that high BMI is an independent risk factor for the development of (symptoms of) MSDs [11-18].

Multiple hypotheses might explain the link between overweight and obesity and musculoskeletal symptoms including, amongst others, increased mechanical demands [19,20] and metabolic factors associated with obesity [19,21]. Increased forces across the joints are likely to play a larger role in the relationship between a high BMI and weight-bearing joints (back and lower extremities), compared to symptoms in non-weight-bearing joints (in the shoulder/neck and upper extremities). For carpal tunnel syndrome (CTS) an increase in upper extremity musculoskeletal symptoms associated with obesity has been attributed to increased adipose tissue in the carpal tunnel, causing median nerve compression [22,23]. Therefore, it seems relevant to make a distinction in different body regions because of potentially different (importance of) risk factors, underlying mechanisms, and natural course of the symptoms.

Weight reduction in overweight and obese workers is assumed to reduce the incidence of musculoskeletal pain [24]. Since overweight and obesity are a growing public health problem, interventions reducing BMI could - if the hypothesised relationship exists - also be an effective primary and secondary prevention strategy for musculoskeletal symptoms.

Epidemiological studies that have demonstrated that high BMI is linked to MSD have not revealed factors that explain this link. Among mechanical factors, adjustment for physical workload could affect the relationship between BMI and MSDs. Occupational physical workload has found to be associated with MSD [25,26]. In a working population, work-related physical load could modify the effect of high BMI on the prevalence of MSD. Our hypothesis is that in workers with high physical workload, the association in weight bearing joints will be increased, through additional physical strain, since overweight and obese individuals experience greater loads on their joints than normal-weight individuals. Analysis of the possible difference in the relationship between high BMI and musculoskeletal symptoms among workers by work-related physical exposure would provide directions for prevention strategies.

The primary research aim of this study was therefore to cross-sectionally investigate the association between BMI and musculoskeletal symptoms in interaction with physical workload. Secondly, since MSDs are of episodic nature, it is of interest to obtain insight into whether high BMI is associated with an increase in occurrence of symptoms in a symptom-free population, or whether high BMI is associated with less recovery from symptoms in a population with symptoms at baseline occurs (or a combination of these options).

## Methods

### Sample/study population

Based on a large working population cohort, we examined BMI in association with prevalence of musculoskeletal symptoms in employees, with adjustment for potential confounders. Additionally, within a subcohort, transitions in musculoskeletal symptoms were longitudinally investigated in relation to BMI.

Data were obtained from The Netherlands Working Conditions Survey (NWCS) [27]. This dataset constitutes of a representative sample of the Dutch workforce in the 15–64 years age group, but excluded self-employed individuals. Each year, 80,000 individuals were sampled from the Dutch working population database by Statistics Netherlands. This database contains information on all jobs that fall under the worker national insurance schemes and are liable to income tax. Sampling was random, except for a 50% over-sampling of employees with lower response rates, namely employees under the age of 25 years and employees with a non-western background. Individuals in the sample received the questionnaire mailed to their home address. After three to four weeks, reminders were sent to those who had not yet responded. Data collection was stopped after two months. To be representative for employees in the Netherlands, the response was weighted for gender, age, sector, ethnic origin, level of urbanization, geographical region and level of education.

The sample was extensively informed about the study in a letter that accompanied the questionnaire. The burden for respondents was low given the topics covered in the questionnaire. Consequently, and in accordance with ethics regulations in the Netherlands, ethical approval was not required for this study.

A total of 44,793 employees completed the NWCS questionnaire in 2008 or 2009 (2008: n = 22,025, 2009: n = 22,768; overall response rate: 28%) and these employees were eligible for the cross-sectional analysis. In addition to the regular annual survey, respondents of the NWCS questionnaire in 2007, who gave consent for being contacted in the future, were invited to respond to follow-up questionnaires in 2008 and 2009 (Netherlands Working Conditions Cohort Study (NWCCS)).

In this cohort, a total of 7,909 completed the NWCCS questionnaire in 2009 (response rate: 35%). Respondents who participated at follow-up were more often higher educated and slightly older than expected based on the NWCS sample. No selective differences were found for the dependent variables BMI and musculoskeletal symptoms. Data retrieved from the NWCCS of these 7,909 respondents were used for the second research aim (i.e., to investigate the transition in musculoskeletal symptoms).

### Measurement of BMI

Self-reported body weight in kilogrammes (kg) and body height in centimetres (cm) were used to determine BMI. BMI was computed as weight (kg)/height (m)^2^. Subsequently, BMI was classified into three categories (normal weight (BMI 18.5-24.9 kg/m^2^), overweight (BMI 25.0-29.9 kg/m^2^), and obese (BMI ≥ 30 kg/m^2^)), which is in accordance with the international classification system of the WHO [28].

#### Measurement of musculoskeletal symptoms

The questions on musculoskeletal symptoms were based on the Dutch Musculoskeletal Questionnaire [29,30]. Employees were asked to rate the occurrence of pain or discomfort in the neck, shoulders, back, arms/elbows, hands/wrists, and lower extremity, in the previous 12 months using 6 questions with five answering categories (‘never’, ‘only once, of short duration’, ‘only once, prolonged’, ‘frequently, of short duration’, ‘frequently and prolonged’). Employees who answered ‘never’ or ‘only once, of short duration’ on all questions were classified as having no musculoskeletal symptoms. Those who answered ‘prolonged’ or ‘frequently’ for one or more locations were classified as having musculoskeletal symptoms overall. Hence, this overall prevalence is reported for any location, in addition to location-specific prevalences for which the responses on neck and shoulders were combined (neck/shoulder), as were those on arms/elbows and hands/wrists (upper extremity).

#### Potential confounders and effect modifiers

Employees were asked questions on current use of force, work in awkward positions, use of vibrating tools (tools, machines or vehicles), and repetitive motions on a 3-point scale (‘never’, ‘yes, occasionally’, yes, regularly’). Employees who answered ‘yes, regularly’ on use of force or work in awkward positions were classified as having high physical workload. Those who answered ‘no, never’ or ‘yes, occasionally’ on both questions were classified as having low physical workload.

Additional potential confounders were gender, age, education (categorized into low, intermediate, and high educational level), contractual working hours (part time/full time), current smoking (yes/no), and physical activity (days a week physically active for at least 30 minutes and of at least moderate intensity). Physical activity was dichotomized as physically active (yes/no) according to the Dutch public health recommendation for moderate intensity physical activity [31]*.*

#### Analysis

For the first research aim, using the weighted cross-sectional data, logistic regression analyses were carried out to investigate the association between BMI and musculoskeletal symptoms. The measure of association was expressed by the Odds Ratio (OR) and its 95% confidence interval (CI). In the categorical analyses involving BMI, the interval 18.5-24.9 was considered as the reference group. In adjusted analysis potential confounders were added to the regression model (full model).

Effect modification was defined as a significant interaction term (p < 0.05) between potential effect modifiers (age, gender, physical workload) and BMI. Analyses were presented stratified for age, gender, or physical workload if the associations between BMI and musculoskeletal symptoms differed based on significant interaction terms.

For the second research aim, using the cohort data (no weighting), the analyses were stratified for respondents without symptoms and those with symptoms in the baseline survey. To determine the difference in the risk of developing symptoms (occurrence) between employees who are overweight and those who are not, outcome was the 12-month incidence of musculoskeletal symptoms. Cases of musculoskeletal symptoms were identified as those who reported frequent or prolonged symptoms at follow-up. To study the influence of BMI on recovery from symptoms, a separate analysis for employees who reported frequent or prolonged symptoms in the last 12 months was performed. Hence, the OR expressed the association between the risk factor at baseline (high BMI) and transition from symptoms to no symptoms, or the reverse, at follow-up.

## Results

### Characteristics and prevalence of symptoms

Table 1 presents the characteristics of the cross-sectional sample. After excluding 865 employees with missing data on BMI (1.9%), and underweight employees (BMI < 18.5; 1.6%), in total 43,221 employees were included in the analysis. Of the employees with normal weight, 50% reported musculoskeletal symptoms within the past 12 months. Musculoskeletal symptoms were reported by 52.3% and 57.6% of the overweight and obese employees, respectively.

**Table 1:** Sample characteristics of musculoskeletal symptoms, demographic, work, and lifestyle-related factors across BMI categories

	**Total**	**‘Normal’ weight**	**Overweight**	**Obese**
**N**	43,221	24,025	14,905	4,291
Symptoms (overall) %	51.6	50.0	52.3	57.6
Neck/Shoulder	30.2	30.0	29.7	33.0
Upper Extremity	20.0	18.3	21.0	26.2
Back	24.0	24.2	23.3	26.0
Lower extremity	24.5	21.4	26.7	34.3
Gender				
Male	54.2	48.0	64.4	53.4
Female	45.8	52.0	35.6	46.6
Age (in years (sd))	40.3(12.1)	37.9(12.3)	43.1(11.2)	43.7(10.9)
Employment				
Full time (> = 36 hrs/wk)	56.5	51.8	63.7	57.0
Part time (<36 hrs/wk)	43.5	48.2	36.3	43.0
Physical workload: repetitive motions				
Regular	33.8	33.1	33.4	38.8
Occasional	22.1	22.3	22.0	21.2
None	44.2	44.6	44.7	40.0
Physical workload: use of vibrating tools				
Regular	9.5	8.0	11.0	12.0
Occasional	9.0	8.2	10.1	9.9
None	81.5	83.8	78.9	78.1
Physical workload: use of force				
Regular	19.2	18.9	19.1	20.6
Occasional	22.5	21.6	23.0	24.9
None	58.3	59.5	57.9	54.4
Physical workload: awkward position				
Regular	10.6	10.0	11.3	11.9
Occasional	25.9	25.6	25.9	27.3
None	63.5	64.4	62.8	60.9
Combined physical workload				
high	22.0	21.7	21.9	23.6
low	78.0	78.3	78.1	76.4
Lifestyle-related factors				
Physically acive (yes)	52.5	54.8	50.3	47.5
Smoking (yes)	27.6	28.1	26.9	27.0

Variables are presented as proportions, with the exception of age (mean (standard deviation)).

### Associations between categories BMI and musculoskeletal symptoms

Table 2 shows the ORs adjusted for age and gender, as well as the ORs after adjustment for all potential confounders (full model). Overall, high BMI (overweight and obesity) was associated with an increased 12-month prevalence of musculoskeletal symptoms. This association was significant for both overweight (OR 1.13, 95% CI: 1.08-1.19) and obesity (OR 1.28, 95% CI: 1.19-1.39) regarding overall musculoskeletal symptoms. Regarding the specific body regions, overweight as well as obesity were associated with increased odds for symptoms. Overweight was associated with upper and lower extremity symptoms (OR 1.10, 95% CI: 1.03-1.17; OR 1.29, 95% CI: 1.21-1.36). Obesity was associated with neck/shoulder (OR 1.12; 95% CI: 1.03-1.21), upper extremity (OR 1.37, 95% CI: 1.25-1.50), back (OR 1.10, 95% CI: 1.01-1.20), and lower extremity symptoms (OR 1.68, 95% CI: 1.55-1.83). Additional (full model) adjustment for employment status (working full time/ part time), level of education, smoking status, physical workload factors, and physical activity level, did not affect the associations.

**Table 2:** Cross-sectional associations between BMI and musculoskeletal symptoms

**Adjusted for age and gender**					
	**Overall**	**Neck/shoulder**	**Upper extremity**	**Back**	**Lower extremity**
Normal weight	1.00	1.00	1.00	1.00	1.00
Overweight	**1.14**	1.04	**1.14**	1.03	**1.31**
	(1.09-1.19)	(0.99-1.09)	(1.08-1.21)	(0.98-1.08)	(1.24-1.37)
Obese	**1.35**	**1.13**	**1.45**	**1.10**	**1.82**
	(1.26-1.44)	(1.06-1.22)	(1.34-1.57)	(1.02-1.19)	(1.69-1.96)
Adjusted for age, gender, smoking, education, contractual working hours(part-time/full-time), use of force, work in awkward positions, use of vibrating tools, repetitive motions, and physical activity					
Normal weight	1.00	1.00	1.00	1.00	1.00
Overweight	**1.13**	1.03	**1.10**	1.02	**1.29**
	(1.08-1.19)	(0.98-1.09)	(1.03-1.17)	(0.96-1.08)	(1.21-1.36)
Obese	**1.28**	**1.12**	**1.37**	**1.10**	**1.68**
	(1.19-1.39)	(1.03-1.21)	(1.25-1.50)	(1.01-1.20)	(1.55-1.83)

Data are presented as Odds Ratios (95% confidence interval), with normal weight as reference category. Significant associations are printed in **bold**.

No effect modification on the association between BMI and musculoskeletal symptoms was found for age or gender. For physical workload, effect modification was found, meaning that the association between BMI and both overall musculoskeletal symptoms and lower extremity symptoms differed between employees with low and high physical workload. This effect modification was not found for neck/shoulder, upper extremity, and back symptoms. Tables 3 and 4 present the model for musculoskeletal symptoms overall and lower extremity symptoms among employees with high as well as low physical workload. The complete model is presented in Additional files 1 and 2. Musculoskeletal symptoms overall and lower extremities were reported significantly more often by obese and overweight employees with low physical workload compared to normal weight employees with low physical workload. For high physical workload, only an association was found for obesity and lower extremity symptoms.

**Table 3:** Prevalence of musculoskeletal symptoms across BMI categories presented separately for high and low combined physical workload

	**Total**	**‘Normal’ weight**	**Overweight**	**Obese**
Physical workload = low	N = 31,622	N = 17,709	N = 10,873	N = 3,040
Overall	15,135	8,156	5,323	1,656
Neck/shoulder	8,621	4,839	2,869	913
Upper extremity	5,349	2,754	1,905	690
Back	6,935	3,944	2,276	715
Lower extremity	6,317	2,982	2,422	913
Physical workload = high	N = 8,897	N = 4,905	N = 3,052	N = 940
Overall	5,713	3,141	1,940	632
Neck/shoulder	3,231	1,778	1,101	352
Upper extremity	2,355	1,202	858	295
Back	2,424	1,347	809	268
Lower extremity	3,220	1,678	1,137	405

**Table 4:** Associations between BMI and Overall musculoskeletal symptoms and lower extremity symptoms stratified for physical workload

**Physical workload = high (n = 8,897)**		
	**Overall**	**Lower extremity**
Normal weight	1.00	1.00
Overweight	0.98	1.07
	(0.88-1.09)	(0.96-1.19)
Obese	1.08	**1.28**
	(0.92-1.28)	(1.09-1.50)
Physical workload = low (n = 31,623)		
Normal weight	1.00	1.00
Overweight	**1.17**	**1.38**
	(1.11-1.24)	(1.29-1.48)
Obese	**1.34**	**1.86**
	(1.23-1.46)	(1.69-2.05)

*Neck/shoulder, upper extremity and back ORs are not presented separately, since no effect modification was found for these body regions. The complete model is presented in Additional file 1.

Data are presented as Odds Ratios (95% confidence interval), with normal weight as reference category, adjusted for age, gender, smoking, education, contractual working hours(part-time/full-time), use of vibrating tools, repetitive motions, and physical activity (full model). Significant associations are printed in bold.

### Effects on the development and recovery of musculoskeletal symptoms

Table 5 presents the effects of BMI on developing musculoskeletal symptoms for employees without symptoms at baseline. The findings on overall symptoms indicated that being obese statistically significantly increased the risk of developing musculoskeletal symptoms during 12-month follow-up (OR 1.37, 95% CI: 1.05- 1.78). Regarding the different body regions, the relationship also existed for lower extremity symptoms for overweight employees (OR 1.35, 95% CI: 1.13-1.61), and for obese employees (OR 2.12, 95% CI: 1.64-2.73). For the upper extremity there was an effect of BMI on occurrence of symptoms for overweight employees (OR 1.22, 95% CI: 1.01-1.46) and for obese employees (OR 1.51, 95% CI: 1.14-1.98). In obese employees the OR was higher than in overweight employees, suggesting a dose–response relationship.

**Table 5:** Occurrence and recovery of musculoskeletal symptoms after 12 months for categories of BMI (overweight and obese), adjusted for age and gender

**Occurrence (from no symptoms to symptoms)**					
	**Overall**	**Neck/shoulder**	**Upper extremity**	**Back**	**Lower extremity**
	**N = 3,663**	**N = 5,071**	**N = 5,591**	**N = 5,085**	**N = 5,410**
Normal weight	1.00	1.00	1.00	1.00	1.00
Overweight	1.17	1.07	1.23	1.13	1.34
	(0.99-1.37)	(0.90-1.28)	(1.01-1.47)	(0.95-1.35)	(1.13-1.60)
Obese	1.37	1.00	1.51	0.94	2.11
	(1.05-1.79)	(0.76-1.33)	(1.14-1.98)	(0.69-1.28)	(1.64-2.72)
**Recovery (from symptoms to no symptoms)**					
	**Overall**	**Neck/shoulder**	**Upper extremity**	**Back**	**Lower extremity**
	**N = 3,841**	**N = 2,086**	**N = 1,378**	**N = 2,005**	**N = 1,667**
Normal weight	1.00	1.00	1.00	1.00	1.00
Overweight	0.97	0.99	0.95	1.06	0.80
	(0.82-1.13)	(0.82-1.22)	(0.75-1.21)	(0.86-1.30)	(0.65-1.00)
Obese	0.76	0.95	0.84	0.99	0.57
	(0.59-0.97)	(0.70-1.30)	(0.59-1.18)	(0.73-1.33)	(0.42-0.78)

Data are presented as Odds Ratios (95% confidence interval), with normal weight as reference category.

The effect of BMI on the recovery from musculoskeletal symptoms after 12 months of follow-up is also presented in Table 5. Employees with obesity recovered less often from musculoskeletal symptoms than employees with normal weight (OR 0.75, 95% CI: 0.59 0.96). This relationship was also found for symptoms in the lower extremity (OR 0.57, 95% CI: 0.42-0.78).

## Discussion

The primary aim of this study was to examine the association between BMI and musculoskeletal symptoms in interaction with physical workload. Overall, high BMI (overweight and obesity) was moderately associated with an increased prevalence of musculoskeletal symptoms in the past 12 months. This association was modified by physical workload. Regarding the second research aim, our longitudinal results showed that for obese employees the association was caused by an increased risk of developing musculoskeletal symptoms during 12-month follow-up as well as less recovery from musculoskeletal symptoms compared to employees with normal weight.

### Lower extremity

Consistent with findings from other studies [31,32] we found the association to be strongest for lower extremity symptoms. The most common joint diseases that cause lower extremity symptoms are osteoarthritis (OA) and rheumatoid arthritis (RA), whereas other causes include musculoskeletal injuries. In the literature it is also suggested that knee pain is a more persistent type of pain, supporting the hypothesis for OA as the cause for symptoms. However, in this cohort lower extremity symptoms were not found to be more persistent than other symptoms in normal weight individuals (data not shown). Obesity had a significant negative effect on recovery from lower extremity symptoms (OR 0.57). Obesity has also, among those with OA as well as in the general population, been found to be associated with disability in mobility [32]. Therefore, biomechanics may explain part of the contribution of the effect of excessive weight on lower extremity symptoms.

#### Upper extremity, and neck/shoulder

The association between high BMI and upper extremity as well as neck/shoulder symptoms could be supporting a non-mechanical hypothesis. This hypothesis is supported by studies showing the association between BMI and the development of OA in non-weight bearing joints, such as the hands [15,33], as well as the link between high BMI and other rheumatic diseases, such as fibromyalgia [34-36]. In a study aimed at weight loss among an obese working population [37] upper extremity symptoms (except for shoulder complaints) decreased with weight loss. In this study it was suggested that many obese subjects use their upper extremities as weight bearing limbs when arising from a seated position, which may account for the increased upper extremity symptoms in obese subjects. However, this explanation is less likely for overweight (non-obese) individuals, for whom in the present study also an association was found. For the upper extremity, an effect of BMI on occurrence of symptoms was found, but not on recovery from symptoms. Overall, the results on upper extremity and neck/shoulder symptoms indicate that most likely metabolic factors are part of the underlying mechanism in the association with high BMI.

#### Back

Yet, in contrast to studies included in a recent meta-analysis [10] no association for overweight and back symptoms in the past 12 months was found. The strength of the association with obesity was modest comparable to the pooled OR from the meta-analysis (1.10 vs. 1.33). Additionally, neither for occurrence nor recovery of back symptoms, overweight or obesity was found to be a risk factor. The finding that workers with high BMI are not at higher risk for developing back symptoms than workers with a normal BMI is in line with a prospective cohort study among health care workers [38].

### Physical workload

It has been argued that for MSD, physical workload as a risk factor itself is more important than BMI [38]. In a study on risk factors for LBP the strength of the association with workload and health behavior (sum of BMI, physical exercise, and smoking) was found to be age-related; workload predicted LBP among those younger than 50 years while health behavior increased the risk among those 50 years or older [39]. In the present study, the association between BMI and MSD differed between employees with low and high physical workload. For musculoskeletal symptoms overall and lower extremity symptoms the association was stronger in those with low physical workload compared to those with high physical workload. No effect modification was found for upper extremity, neck/shoulder, or back symptoms. Contradictory to our hypothesis, the association of BMI and lower extremity symptoms was found to be weaker for employees with higher physical workload. This implies that the association may not be simply due to weight related increased excessive loading of the joint. Based on these results, it is possible that for employees with high BMI and high physical workload, muscle mass around the knee joint is protective for the development of MSD. Weakness of the quadriceps have been considered a primary risk factor for knee pain and disability in persons with OA [40]. There is evidence to hypothesize that muscle mass protects the knee joint, with increased muscle strength protecting against incidence knee OA (greater joint stability and cartilage volume) [41]. Further support for this explanation comes from research on functional limitations as a consequence of obesity. Increased body mass can have negative influences on the control of postural stability and locomotion [42]. Poorer balance was found to be associated with higher pain in the presence of less muscle strength [43]. Support for this notion also comes from literature that shows that muscle strengthening, as a part of treatment, reduces disability from MSD [44-46]. In addition, loss of muscle mass as well as central obesity (not BMI) were found to be possible risk factors for LBP [47].

#### Methodological strengths, and limitations

The main strength of this study is the large sample that included a nationally representative sample of the Dutch workforce. This provided sufficient statistical power to examine overweight and obesity in association with musculoskeletal symptoms in employees for physical workload categories, as well as different locations of symptoms.

Some limitations should be considered as well. The study is conducted in a worker population, and when translating the results to the general population, the healthy worker (survival) effect should be taken into account. By exploring the association in a working population it is possible that workers, who have severe MSD, are no longer employed or change to work with lower exposure.

In the analysis the association was controlled for several potential confounding factors, however some potential psychosocial confounders, for instance stress, anxiety or depression disorders, were not measured, and consequently could not be controlled for.

The use of self-reported measures could be considered a limitation as they are susceptible to possible bias. Self-reported workload might be biased by the presence of symptoms. In workers performing the same job, workers with MSD reported higher exposure rates than workers without MSD [48]. However, in the present study self-reported workload was used to identify high exposure from low exposure, with highly contrasting jobs and working conditions. Misclassification in categories BMI, as a result of underreporting of body weight, could hypothetically lead to underestimation of the association with MSD. Furthermore, BMI as a measure does not discriminate adipose from non-adipose body mass, nor does it indicate the distribution of body fat. Stronger associations with abdominal obesity than general obesity and LBP were found in population-based studies [49]. Additional measurements of fat distribution would provide insight in possible factors of the mechanism of the effect (posture, loading etc.).

For the first research question the cross-sectional design prevents conclusions of causality. Weight gain may also occur as a consequence of musculoskeletal pain and physical inactivity. Therefore, the measured BMI may not in all cases reflect BMI before the onset of symptoms. Weight gain following the onset of symptoms (e.g. because of reduced physical activity due to symptoms) may have caused overestimation of the associations. For the second research aim prior history (>1 year) of symptoms are not taken into account. In this study, the definition of the symptom-free population was based on reporting no symptoms in the previous 12 months, which is considered long enough to exclude those with frequently recurring symptoms. Selection bias may have occurred as a result of the low response rate. Persons lost to follow-up were younger and less often highly educated than those who responded to the follow up questionnaire. However, no difference was found for BMI and dependent variables musculoskeletal symptoms between those lost to follow-up and respondents.

## Conclusions

In summary, in this study, BMI was associated with musculoskeletal symptoms, in particular symptoms of the lower extremity. Furthermore, the association was stronger for employees with low physical workload compared to those with high physical workload. Compared to employees with normal weight, obese employees had higher risk for developing symptoms as well as less recovery from symptoms. This study supports the role of biomechanical factors for the relationship between BMI and MSD in the lower extremity.

With an increasing public health problem resulting from overweight and obesity, and since overweight and obesity are a preventable or modifiable risk factor, these findings give directions to prevention strategies. The risk on musculoskeletal health problems should be taken into account in primary as well as secondary prevention strategies. To address MSD in a worker population, weight loss or preventing weight gain strategies alone may not be sufficient. The physical consequences of loading of major structures, particularly in the lower extremity as a consequence of overweight and obesity deserve attention.

## Competing interests

The authors declare that they have no competing interests.

## Authors’ contributions

LV conceived of the study, performed the statistical analysis, and drafted the manuscript. LK was responsible for the coordination of the data collection. LK, EV, KOH, AB, and PB were involved in the design of the study, and contributed to the interpretation of the data. All authors contributed to the manuscript by reading and correcting draft versions. All authors read and approved the final manuscript.

## Pre-publication history

The pre-publication history for this paper can be accessed here:

http://www.biomedcentral.com/1471-2474/14/238/prepub

## Supplementary Material

Additional file 1 Associations between BMI and musculoskeletal symptoms with normal weight and low workload as reference category. Click here for file

Additional file 2 Univariable and multivariable associations between BMI, workload, and BMI* workload and musculoskeletal symptoms. Click here for file
