# Supplementary material for: The relation between body mass index and musculoskeletal symptoms in the working population
Source: BMC Musculoskelet Disord. 2013 Aug 12;14:238. doi: 10.1186/1471-2474-14-238 (PMC3751130; doi:10.1186/1471-2474-14-238)
Supplement: Additional file 1 — Associations between BMI and musculoskeletal symptoms with normal weight and low workload as reference category. [file 1471-2474-14-238-S1.doc]

Additional file 1

Associations between BMI and Overall musculoskeletal symptoms and Lower Extremity symptoms. Data are presented as Odds Ratios (95% confidence interval), **with normal weight and low workload as reference category**, adjusted for age, gender, smoking, education, contractual working hours(full-time/part-time), and physical activity.

|  |  |  |
| --- | --- | --- |
|  | Overall | Lower extremity |
| Normal weight and low workload | 1.00 | 1.00 |
| Normal weight and high workload | 2.22 (2.06 - 2.39) | 2.50 (2.31-2.71) |
| Overweight and low workload | 1.18 (1.11 - 1.24) | 1.37 (1.29-1.47) |
| Overweight and high workload | 2.21 (2.02 - 2.42) | 2.78 (2.53-3.06) |
| Obese and low workload | 1.36 (1.25 - 1.48) | 1.88 (1.70 – 2.07) |
| Obese and high workload | 2.47 (2.12 – 2.89) | 3.29 (2.82-3.82) |
