# Supplementary material for: The relation between body mass index and musculoskeletal symptoms in the working population
Source: BMC Musculoskelet Disord. 2013 Aug 12;14:238. doi: 10.1186/1471-2474-14-238 (PMC3751130; doi:10.1186/1471-2474-14-238)
Supplement: Additional file 2 — Univariable and multivariable associations between BMI, workload, and BMI* workload and musculoskeletal symptoms. [file 1471-2474-14-238-S2.doc]

Additional file 2

Univariable and multivariable associations between BMI, workload, and BMI*workload and musculoskeletal symptoms. Data are presented as Odds Ratios (95% confidence interval), mutually adjusted, and adjusted for age, gender, smoking, education, contractual working hours(part-time/full-time), use of vibrating tools, repetitive motions, and physical activity.

| Univariable model | | | | | |
| --- | --- | --- | --- | --- | --- |
|  | Overall | Neck/shoulder | Upper extremity | Back | Lower extremity |
| BMI |  |  |  |  |  |
| Normal weight | 1.00 | 1.00 | 1.00 | 1.00 | 1.00 |
| Overweight | **1.13**  (1.08-1.19) | 1.03  (0.98-1.09) | **1.10**  (1.03-1.17) | 1.02  (0.96-1.08) | **1.29**  (1.21-1.36) |
| Obese | **1.28**  (1.19-1.39) | **1.12**  (1.03-1.21) | **1.37**  (1.25-1.50) | **1.10**  (1.01-1.20) | **1.68**  (1.55-1.83) |
| Combined workload |  |  |  |  |  |
| Low physical workload | 1.00 | 1.00 | 1.00 | 1.00 | 1.00 |
| High physical workload | **1.77**  (1.66-1.88) | **1.48**  (1.39-1.58) | **1.46**  (1.36-1.57) | **1.37**  (1.28-1.47) | **1.84**  (1.72-1.97) |
| Multivariable model | | | | | |
| BMI |  |  |  |  |  |
| Overweight | **1.18**  (1.12-1.24) | 1.04  (0.98-1.11) | **1.10**  (1.02-1.18) | 1.03  (0.96-1.10) | **1.39**  (1.30-1.48) |
| Obese | **1.34**  (1.23-1.46) | **1.14**  (1.04-1.25) | **1.41**  (1.27-1.56) | **1.11**  (1.00-1.23) | **1.86**  (1.69-2.05) |
| High physical workload | **1.92**  (1.77-2.08) | **1.52**  (1.40-1.65) | **1.49**  (1.36-1.64) | **1.39**  (1.27-1.51) | **2.11**  (1.93-2.30) |
| BMI*combined workload | P=0.003 | P=0.610 | P=0.600 | P=0.950 | P <0.00001 |
| Overweight*workload | **0.84**  (0.74-0.94) | 0.97  (0.85-1.09) | 0.98  (0.85-1.12) | 0.98  (0.86-1.12) | **0.77**  (0.68-0.88) |
| Obese*workload | **0.81**  (0.67-0.98) | 0.91  (0.76-1.11) | 0.90  (0.73-1.10) | 0.98  (0.80-1.20) | **0.69**  (0.57-0.83) |
